# Biological basis for novel mesothelioma therapies

**DOI:** 10.1038/s41416-021-01462-2

**Published:** 2021-07-05

**Authors:** Joanna Obacz, Henry Yung, Marie Shamseddin, Emily Linnane, Xiewen Liu, Arsalan A. Azad, Doris M. Rassl, David Fairen-Jimenez, Robert C. Rintoul, Marko Z. Nikolić, Stefan J. Marciniak

**Affiliations:** 1 Cambridge Institute for Medical Research, Keith Peters Building, Cambridge Biomedical Campus, University of Cambridge, Cambridge, UK; 2 UCL Respiratory, Division of Medicine Rayne Institute, University College London, London, UK; 3 Wellcome Sanger Institute, Wellcome Trust Genome Campus, Saffron Walden, UK; 4 Adsorption & Advanced Materials Laboratory, Department of Chemical Engineering and Biotechnology, University of Cambridge, Cambridge, UK; 5 Department of Histopathology, Royal Papworth Hospital NHS Foundation Trust, Cambridge, UK; 6 Department of Oncology, University of Cambridge, Cambridge, UK; 7 Department of Thoracic Oncology, Royal Papworth Hospital NHS Foundation Trust, Cambridge, UK; 8 Department of Medicine, University of Cambridge, Addenbrooke’s Hospital, Cambridge, UK

**Keywords:** Mesothelioma, Mechanisms of disease

## Abstract

Mesothelioma is an aggressive cancer that is associated with exposure to asbestos. Although asbestos is banned in several countries, including the UK, an epidemic of mesothelioma is predicted to affect middle-income countries during this century owing to their heavy consumption of asbestos. The prognosis for patients with mesothelioma is poor, reflecting a failure of conventional chemotherapy that has ultimately resulted from an inadequate understanding of its biology. However, recent work has revolutionised the study of mesothelioma, identifying genetic and pathophysiological vulnerabilities, including the loss of tumour suppressors, epigenetic dysregulation and susceptibility to nutrient stress. We discuss how this knowledge, combined with advances in immunotherapy, is enabling the development of novel targeted therapies.

## Background

Mesothelioma is a malignancy primarily of the thoracic and abdominal linings that affects 2600 individuals in the UK annually with a median survival of about 1-year post-diagnosis [1]. It develops from cells of the mesothelium, a serous membrane that lines the coelomic cavities (Box 1), frequently as a result of asbestos exposure, although other potential triggers of mesothelioma include engineered long, straight, carbon nanotubes, genetic predisposition and radiation therapy. In the case of asbestos, inhaled microscopic fibres migrate through the lung to the pleural space where they can persist for decades, activating mitogenic and inflammatory pathways. Local generation of reactive oxygen species by the asbestos fibres appears to cause DNA damage, triggering malignant transformation.

**Fig. 1:**
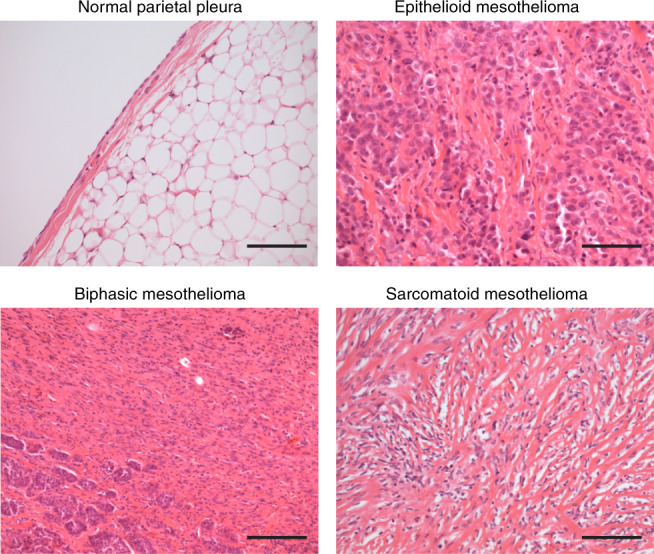
The histopathological classification of malignant mesothelioma. Images of haematoxylin and eosin (H&E) stained normal pleura (×100), epithelioid (×100), sarcomatoid (×100) and biphasic (×100) mesothelioma subtypes, indicating the presence of flat, cuboidal cells in epithelioid mesothelioma as well as spindle cells and abundant stroma in sarcomatoid mesothelioma. Scale bar = 200 µm. The images were provided by Royal Papworth Hospital Research Tissue Bank.

Malignant pleural mesothelioma accounts for around 80% of reported cases of mesothelioma, followed by peritoneal (∼20% cases) and tunica vaginalis (∼1% cases) mesotheliomas. This review focuses on the most common pleural mesothelioma. The three main histological subtypes are epithelioid (50–70% of cases), sarcomatoid (10–20% of cases) and mixed or biphasic (30% of cases) [2] [Fig. 1]. Epithelioid mesothelioma cells often resemble benign, reactive mesothelial cells—which exist as flat or cuboidal forms—with varying degrees of atypia. By contrast, sarcomatoid mesothelioma consists of spindle cells, while biphasic mesothelioma has both epithelioid and sarcomatoid elements. Typically, stroma is abundant and often comprises the majority of the tumour mass. The subtypes represent a spectrum of trans-differentiation with the sarcomatoid phenotype being driven by epithelial–mesenchymal transition (EMT); while epithelioid mesothelioma involves mesenchymal–epithelial transition (MET) [3]. Although EMT is not routinely used for diagnosis, it might be relevant to histological subtyping, with downregulation of EMT markers (e.g. cadherin, c-MYC and vascular endothelial growth factor receptor (VEGFR2)) and upregulation of EMT transcription factors (e.g. Slug, Twist, ZEB1 and ZEB2) observed in sarcomatoid mesothelioma [4, 5]. Accordingly, epithelial markers E-cadherin, β-catenin and cytokeratins 5/6 are abundantly expressed in epithelioid mesothelioma and progressively lost in biphasic and sarcomatoid subtypes [5].

The histological subtypes remain the main prognostic parameter for mesothelioma patients [6]: sarcomatoid mesothelioma has the worst prognosis, with a median survival of 4 months, compared with 13.1 months and 8.4 months for epithelioid and biphasic mesotheliomas, respectively [1, 5]. Histology can be combined with age, gender, probability of diagnosis and leukocyte count in the validated European Organisation for Research and Treatment of Cancer (EORTC) composite score to help predict survival [7, 8]. Other potential prognostic markers arising from studies of cohorts of patients with mesothelioma include the ER stress marker CHOP (C/EBP homologous protein) [8], monocarboxylate transporter 4 (MCT4) [9], CD31 (a stromal marker) [10], periostin and phosphatase and tensin homologue (PTEN) (EMT-related molecules) [11].

Owing to its insidious presentation, only a minority of patients are diagnosed with early stage 1A or 1B mesothelioma that is amenable to surgery. Although cytoreduction by pleurectomy-decortication or radical extra-pleural pneumonectomy (EPP) has been advocated [2], the results have been disappointing [12]. For inoperable cases, palliative chemotherapy is the current standard of care in Europe [13], with a combination of pemetrexed and cisplatin increasing median survival by 3 months [13]. The development of resistance to chemotherapy is a major problem, with almost 50% of mesotheliomas showing resistance to the treatment [13]. An important unmet need therefore exists for effective therapy for this cancer. Understanding the biology of mesothelioma could identify new therapeutic approaches.

Here, we discuss the genetic and cellular vulnerabilities identified in mesothelioma, and the tumour immune landscape, which together provide a rational for the development of novel treatments. We provide a comprehensive summary of previous and current clinical trials.

Box 1 Biology of healthy mesothelium The mesothelium is a serous membrane that lines the coelomic cavities. It is derived from embryonic mesoderm and comprises the pleura, pericardium, peritoneum, tunica vaginalis testis and tunica serosa uteri. During development, the lateral plate mesoderm divides into layers, one of which combines with ectoderm to become the somatopleure, which forms the body wall and parietal pleura; another combines with endoderm to become the splanchnopleure, giving rise to the coelomic organs and visceral pleura.

Chimera experiments using chick embryos have revealed that most mesothelium derives from an organ-intrinsic mesothelial precursor [215]. In humans, the pleura forms between the fifth and sixth embryonic weeks, before the submesothelial connective tissue. Lineage tagging suggests that mesothelial precursors migrate into the mesenchyme, where they undergo mesothelial–mesenchymal transition (MMT) contributing to bronchial smooth muscle, vascular smooth muscle and fibroblasts [216, 217]. Wilms’ tumour 1 (WT1) is a transcription factor that serves as a mesothelial marker owing to its relatively limited expression in healthy adult tissues. WT1–Cre marks cells of pleural mesothelial origin in mice and has revealed that WT1^+^ cells co-localise with α-smooth muscle actin (SMA)^+^ cells in pulmonary vessels. Migration of WT1^+^ cells into the parenchyma was imaged by time-lapse microscopy in fetal murine lung [216]. In healthy adults, WT1 appears to control the integrity of pleural membranes by preventing MMT [218].

The pleural cavity is the potential space between adjacent visceral and parietal pleurae, which contains a thin layer of fluid and allows low friction sliding between the two serous membranes [Figure 4]. The pleura itself is only 40 μm thick and consists of five layers: a monolayer of mesothelial cells on a basal lamina; a superficial elastic layer; a collagen-rich layer containing vessels, nerves and immune cells; and a deep fibroelastic layer. The deep layer is tightly adherent to the underlying structures e.g. muscle, rib or lung parenchyma. Kampmeier foci on the mediastinal pleura contain aggregates of macrophages and lymphocytes, which are involved in immune surveillance and phagocytosis of irritants and pathogens.

Mesothelial cells can exist in flat or cuboidal forms. Cell–cell contacts towards their apical surfaces regulate pleural permeability and maintain cell polarity; N-cadherin rather than epithelial E-cadherin is found in mesothelial adherens junctions. Mesothelin is a 40-kDa glycoprotein expressed on mesothelial cells, which mediates cell adhesion partially through its interaction with mucin CA125/MUC16 [219]. Its purpose remains ambiguous as no abnormalities were detected in mesothelin-knockout mice [220].

Pleural fluid is secreted predominantly from the apical zones of the parietal pleura by filtration from capillaries. Numerous microvilli provide a large surface area for secretion and absorption. In addition, primary cilia detect friction and inflammatory cytokines, and so are important in mesothelial repair. When these roles in lubrication and inflammation are perturbed, fibrosis and adhesions can occur.

## Hallmarks of mesothelioma of potential therapeutic relevance

Targeting intrinsic features of cancer, such as genetic instability, unchecked growth, aberrant energetics, enhanced angiogenesis, and immune evasion, suggest a variety of therapeutic options in mesothelioma. We therefore begin by assessing this biology.

### Genetic alterations in mesothelioma

**Fig. 2:**
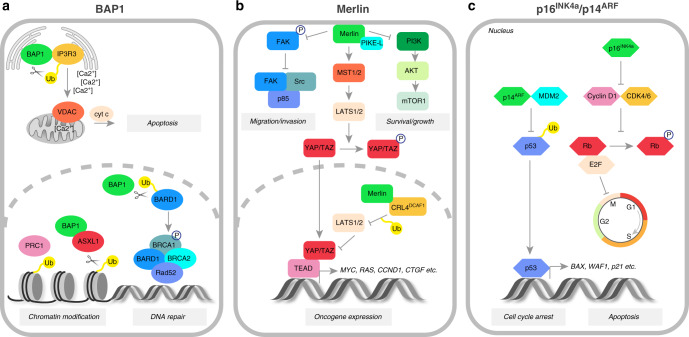
Tumour suppressor functions of BAP1, Merlin and p16^INK4a^/p14^ARF^ proteins. **a** In the endoplasmic reticulum (ER), BAP1 deubiquitinates and stabilises the type-3 inositol-1,4,5-trisphosphate receptor (IP3R3), controlling the Ca^2+^ flux into mitochondria and the subsequent release of cytochrome *c* and apoptosis. The nuclear BAP1 activity leads to BRCA1–BARD1-complex-dependent DNA repair as well as chromatin modification through histone deubiquitination. **b** At the plasma membrane, Merlin inhibits promigratory and prosurvival signalling cascades, including those mediated by focal adhesion kinase (FAK)–Src and phosphatidylinositol 3-kinase (PI3K)–AKT–mammalian target of rapamycin (mTOR)1 pathways. It also activates the mammalian Hippo pathway, which results in the phosphorylation of YAP/TAZ transcription co-activators, precluding their nuclear translocation and the TEA domain (TEAD)-dependent expression of numerous oncogenes. **c** p14^ARF^ promotes cell-cycle arrest and apoptosis by preventing p53 degradation, while p16^INK4a^ inhibits cell-cycle progression by binding and inactivating CDK4/6 protein and the downstream effector, retinoblastoma (Rb) protein.

Mesothelioma has a modest mutational burden compared with other cancers [14–16]. Nevertheless, a number of key tumour suppressor genes are frequently affected, including those encoding cyclin-dependent kinase inhibitor 2A (*CDKN2A*), BRCA1-associated protein 1 (*BAP1*) and neurofibromin 2 (*NF2*) [17–19] [Fig. 2]. The importance of alterations in these genes is supported by whole-exome sequencing and somatic copy number alteration (SCNA) analyses [15, 16, 20, 21].

#### BAP1

The importance of BAP1 genetics in the development and prognosis of mesothelioma has been extensively reviewed elsewhere [22–24]. In humans, germline mutations in *BAP1* predispose to malignant mesothelioma [25], and together with other pathogenic mutations linked to cancer accounts for 12% of all cases [26]. Remarkably, however, patients with mesothelioma who carry germline mutations of *BAP1* have a significantly better prognosis compared with those with sporadic disease, although the mechanism of this is unknown [26, 27]. BAP1 is an important regulator of interactions between genes and the environment [28], and loss of *BAP1* enhances the susceptibility of fibroblasts and mesothelial cells to ionising or UV irradiation, and to asbestos [29], which contributes to the development of asbestos-induced mesothelioma in vivo [30, 31]. The *BAP1* gene product is a deubiquitinating enzyme that plays a key role in the nucleus in the cell cycle, cell death and the DNA damage response [22, 23]; while in the cytoplasm, it triggers apoptosis by regulating the release of calcium ions from the endoplasmic reticulum through its interaction with the receptor for inositol 1,4,5-trisphosphate, IP3R3 [29]. BAP1 is also involved in the epigenetic regulation of many genes via polycomb repressor complex 2 (PRC2), which has potential therapeutic relevance [22], as the loss of BAP1 promotes sensitivity to PRC2 inhibitors, which block tumour growth and invasion [32]. One such drug, tazemetostat, is currently in phase II clinical trials for mesothelioma (NCT02860286).

#### NF2

*NF2* encodes Merlin, which negatively regulates receptor-dependent mitogenic signalling, and downstream phosphatidylinositol 3-kinase (PI3K)–AKT activity, and activates Hippo pathway [33]. Although the loss of NF2 has been suggested to drive mesothelioma carcinogenesis [34], additional mutations are necessary as demonstrated by murine models that require combined deletions such as *NF2;INK4a/ARF* or *NF2;p53* [35]. Merlin controls the expression of oncogenic genes by inhibiting the E3 ubiquitin ligase CRL4^DCAF1^, to stabilise large tumour suppressor kinase 1/2 (LATS1/2), which in turn inhibits the transcriptional co-activators YAP and TAZ, two downstream effectors of the Hippo pathway [36]. Mutations of Hippo pathway components, such as *LATS1* and *LATS2*, have been observed in many mesothelioma specimens with aberrant YAP activation [16, 21]. Furthermore, co-inactivation of *LATS2* and *NF2* in mesothelioma cell lines triggers the loss of cell–cell contact inhibition, dysregulation of Hippo and mammalian target of rapamycin (mTOR) signalling, and correlates with higher sensitivity to inhibitors of the PI3K–AKT–mTOR pathway [37]. Targeting the activation of YAP commonly observed in mesothelioma [38] either by inhibiting Rho-associated kinase (ROCK), a downstream target of YAP, or by disrupting the interaction of YAP with TEA domain (TEAD) transcription factors using verteporfin—impedes mesothelioma cell proliferation and invasion in vitro [39]. Interestingly, Hippo signalling appears to stabilise growth arrest and DNA damage 34 (GADD34) protein, resulting in increased YAP phosphorylation and retention in the cytoplasm, as GADD34 directs protein phosphatase 1 (PP1) away from YAP [40]. This might be particularly relevant to mesothelioma, as GADD34 expression correlates with mesothelial cell differentiation and is lost in more aggressive subtypes [8].

#### CDKN2A

*CDKN2A* encodes two cell-cycle regulators, p16^INK4a^ and p14^ARF^ [17, 41]. p16 ^INK4a^ inhibits cyclin-dependent kinase (CDK)4 and CDK6-dependent phosphorylation of retinoblastoma protein (RB), whereas p14^ARF^ prevents p53 degradation by Mdm2 [41]. Mutations in *TP53* occur in less than 10% of mesotheliomas [15, 21] but correlate with worse survival [16]. Given that *CDKN2A* is deleted in approximately 45% of mesothelioma cases [15], palbociclib, a CDK4/6 kinase inhibitor, has been tested for its ability to induce cell-cycle arrest and senescence in mesothelioma [42]. Treatment with palbociclib led to AKT phosphorylation, but was shown to induce synergistic inhibition of cell proliferation when combined with PI3K/AKT/mTOR inhibitors.

#### Additional alterations

The majority of studies have focused on the more common epithelioid mesothelioma, with relatively few reports pertaining to the rarer, but more aggressive, sarcomatoid subtype. A report in 2020 showed that loss of *PTEN* or *TP53* promotes the development of non-epithelioid mesotheliomas, with activation of PI3K and MEK–ERK/MAPK [43]. Accordingly, combined pharmacological inhibition of MEK using selumetinib and PI3K using AZD8186 inhibited tumour growth and increased survival in mice [43], pointing towards a potential novel targeted strategy for sarcomatoid mesothelioma. Other profiling studies have identified potential therapeutic vulnerabilities in this rare subset of mesotheliomas [44–46]. For instance, LOXL2 (an EMT marker) and VISTA (an immune checkpoint) were found to be overexpressed in tumours with sarcomatoid-like characteristics [16, 44], while differential expression of EMT-related genes identified a subgroup of mesothelioma patients with poor prognosis [45]. Another molecular subgroup displaying sarcomatoid features appears to be more sensitive to drugs inhibiting Wee1, a component of the G2/M cell-cycle checkpoint, and ROCK [44].

#### Epigenetic dysregulation in mesothelioma

Post-translational modification of histones and methylation of DNA are often altered in cancers [47] and, in mesothelioma, hypermethylation of tumour suppressor gene (*CDK2NA*, *APC*, *CCND2* and *RASSF1*) promoters and upregulation of DNA methyltransferases (DNMTs) have been reported [48], while mutations in histone methyltransferases (*SETDB1* and *SETD2*) are often found in patient samples [16, 21, 49]. These observations provided a rationale for targeting histone deacetylases (HDACs) using specific inhibitors (HDACi), which have multiple anticancer effects: they antagonise the cell-cycle and angiogenesis, promote apoptosis and exert anti-inflammatory activity [50, 51]. A phase I trial of vorinostat (an inhibitor of HDAC1,2,3 and 6) [52] in 13 patients with mesothelioma showed a partial response in two patients [53]. Unfortunately, a subsequent phase III trial in 650 patients with mesothelioma demonstrated no improvement in overall survival (OS) [54]. Newer HDACi, such as trichostatin A and its analogues, show promising activity in combination with the DNMT inhibitor decitabine plus immunotherapy [55]. In vitro studies suggest that the loss of BAP1 affects the sensitivity of mesothelioma cells to HDACi [56], indicating that stratification of patients might help to identify a responsive subgroup. Furthermore, decitabine might also prove anti-proliferative in mesothelioma through the upregulation of p21, independent of its effect on DNMTs [57].

Loss of tumour suppressive microRNAs that control cell growth, migration and apoptosis is often observed in mesothelioma [58]. Several preclinical studies have reported that restoring such microRNAs using synthetic mimics can confer anti-tumour activity; examples include miR-15/16 [59] and miR-34 [60] family members, as well as miR-1 [61], miR-31 [62] and miR-145 [63]. Moreover, miR-16-based mimics that target epidermal growth factor receptor (EGFR) have shown acceptable safety and preliminary activity in a phase I study in 26 mesothelioma patients [64].

### Mesothelioma cell proliferation and motility

#### Focal adhesion kinase

Focal adhesion kinase (FAK) is a tyrosine kinase that regulates cancer cell survival, proliferation, migration and invasiveness [65]. In vitro studies have shown the upregulation of FAK in many mesothelioma cell lines, whereas FAK inhibition suppresses cell proliferation and the ability to form anchorage-independent colonies [66]. Accordingly, the FAK inhibitor BI853520 abrogates the growth of orthotopic tumours in vivo [67]. As mentioned above, the tumour suppressor Merlin is frequently inactivated in mesothelioma. Since Merlin can inhibit cell migration and invasiveness by attenuating FAK phosphorylation [68], low Merlin expression was predicted to increase sensitivity to FAK inhibitors [69] and, indeed, a phase I clinical trial of the FAK inhibitor GSK2256098 improved the median progression-free survival (PFS) of patients lacking detectable Merlin [70]. This result was supported by data from a phase Ib study of 34 patients treated with GSK2256098 plus the MEK inhibitor trametinib (to target probable mitogen-activated protein kinase pathway (MAPK) activation), in which longer PFS was reported for Merlin-negative tumours compared with Merlin-positive tumours [71]. Based on these data, a phase II trial of defactinib, an orally bioavailable FAK inhibitor, recruited 344 patients with mesothelioma and low levels of Merlin but, unfortunately, no improvement in disease outcome was observed [72]. Of note, GSK2256098 is selective for FAK while defactinib targets FAK and Pyk2, and so target specificity might have been a confounder.

#### c-MET

c-MET, a receptor tyrosine kinase important in cell proliferation and motility, is also overexpressed in mesothelioma [73]. In vitro and in vivo studies have revealed that targeting c-MET with tivantinib (ARQ 197) together with PI3K inhibition suppresses cell motility, growth and the development of tumours [74]. A phase I/II clinical trial of tivantinib in combination with pemetrexed and carboplatin/cisplatin is underway in patients with mesothelioma or non-small cell lung cancer (NCT02049060).

### Tumour nutrient availability

#### Angiogenesis

Most solid cancers promote angiogenesis to support their growth [75]. Angiogenic signalling is important for mesothelioma growth [76], and high expression levels of pro-angiogenic signalling molecules, such as VEGF, fibroblast growth factor (FGF)-1, transforming growth factor-β (TGF-β), platelet-derived growth factor (PDGF) and PDGF receptor-β, have been reported in mesothelioma tissue and many cell lines [76–78]. Furthermore, a high density of tumour microvessels predicts poor survival in patients with malignant mesothelioma [79]. Consequently, inhibitors of angiogenesis have been investigated for potential treatment in patients with mesothelioma [80–82]. Thalidomide, a potent inhibitor of angiogenesis in other cancers [83, 84], unfortunately provided no benefit in a phase III trial in mesothelioma [85]. Cediranib, a tyrosine kinase inhibitor targeting VEGFR 1–3, c-Kit and PDGFR-β [86], seemed effective as a second-line agent in one phase II trial [82] and, when combined with chemotherapy, appeared to improve survival in treatment-naïve patients, but significant toxicity precluded its further development [87]. Another tyrosine kinase inhibitor, nintedanib, which targets VEGFR 1–3, FGFR 1–3, PDGFR α/β and Src-family members, increased PFS and OS when combined with pemetrexed/cisplatin in a phase II trial [81] but, unfortunately, this promising result failed to be validated in a subsequent phase III study [80]. More encouraging results have been achieved using bevacizumab (Avastin), a humanised anti-VEGFA monoclonal antibody, which demonstrated efficacy in combination with standard of care in many cancers [88, 89] including mesothelioma [90]. In a large phase III trial (Mesothelioma Avastin Cisplatin Pemetrexed Study, MAPS) of 448 patients, median OS was increased from 16.1 to 18.8 months [90], which resulted in bevacizumab being listed in the American National Comprehensive Cancer Network guidelines as a potential first-line treatment for unresectable mesothelioma [91]; however, at the time of writing, the manufacturer appears not to be pursuing its licensing for bevacizumab for the treatment of mesothelioma in Europe.

#### ER stress and nutrient stress

Mesothelial cells produce high quantities of cell-surface glycoproteins, most likely to help lubricate the pleural cavities, and therefore depend on the presence of a functional endoplasmic reticulum (ER) in which to fold these proteins. Increased protein misfolding, which might well occur in response to increased demands for protein secretion, is known to cause ER stress signalling, which has emerged as a key adaptive mechanism supporting cancer progression and resistance to treatment [92]. The expression of ER-stress-responsive GADD34 is lower in sarcomatoid mesothelioma than in epithelioid mesothelioma, while high levels of ER-stress-dependent transcription factor C/EBP homologous protein (CHOP) predict poor prognosis [8]. Increased expression of the ER chaperone BiP (GRP78) was reported in mesothelioma cell lines [93] and in patient samples [94]. Consequently, modulation of the ER stress response has been investigated in mesothelioma in preclinical models. For instance, bortezomib, a proteasome inhibitor, and epigallocatechin-3-gallate, a green tea polyphenol, trigger ER-stress-dependent cell death in mesothelioma cell lines [93, 95]. Moreover, HA15, an ER stress inducer that specifically targets BiP [96], exacerbates pre-existing high ER stress levels in mesothelioma cells to induce cell death and impairs mesothelioma cell growth in patient-derived xenografts in a CHOP-dependent manner [94].

Nutrient stress can also affect mesothelioma growth. For example, inhibition of a key component of the glycolytic pathway, 6-phosphofructo-2-kinase/fructose-2,6-biphosphatase 3 (PFKFB3), results in anti-tumour activity in mesothelioma, with death occurring at least in part via the induction of ER stress [97]. Mesothelioma cells are often arginine auxotrophic owing to the loss of argininosuccinate synthetase 1 (ASS-1) [98], a phenomenon that led to a phase II trial of the arginine-degrading agent PEGylated arginine deiminase (ADI-PEG20). ADI-PEG20 treatment resulted in depletion of circulating arginine and improved PFS in mesothelioma patients [99]. Encouragingly, the TRAP phase I trial of ADI-PEG20 combined with pemetrexed/cisplatin chemotherapy revealed 94% disease control in biphasic and sarcomatoid subtypes [100]. The subsequent phase II/III ATOMIC-Meso global trial of 386 patients with non-epithelioid mesothelioma is recruiting, with a completion date of June 2022 (NCT02709512).

### The immune landscape of mesothelioma

Tumour-infiltrating lymphocytes (TILs), macrophages and natural killer (NK) cells are detectable in mesothelioma tissue [101, 102], while mesothelioma can promote an immunosuppressive environment through regulatory T (T_REG_) cells [102] and M2 polarised macrophages [103]. Whereas infiltration with cytotoxic CD8^+^ lymphocytes correlates with better patient prognosis [101, 104], high levels of ‘tumour promoting’ M2 macrophages predict shorter patient survival in patients with pleural mesothelioma [105]. Interestingly, as reported for peritoneal mesothelioma, the histological subtypes might show distinct immune signatures, with TILs and Th-1 polarised T cells predominantly present in epithelioid mesothelioma, and myeloid cell infiltrating all subtypes [106]. In pleural mesothelioma, increased expression of immune checkpoint programmed death ligand-1 (PD-L1) and the presence of sarcomatoid components is associated with increased stromal TILs, which, if characterised by high CD8^+^ and low CD4^+^, predict poor prognosis [107]. Such knowledge is driving the development of immunomodulatory therapies for mesothelioma.

## Immunotherapeutic strategies for mesothelioma

Rare cases of spontaneous regression of mesothelioma have been attributed to immune responses [108], suggesting that immunotherapy might be efficacious in this cancer type, similar to the case in several other cancers [109–111].

### Immune checkpoint inhibitors

The tumour-killing immune response can be inhibited by cancer cells that express regulators of immune checkpoints such as cytotoxic T-lymphocyte antigen 4 (CTLA-4), T cell immunoglobulin mucin-3 (TIM-3) and PD-L1 [112]. PD-L1, for example, is expressed in up to 29% of mesotheliomas [113] and is associated with poor survival [114]. Inhibition of these checkpoints has proved useful in many cancers and trials have already investigated the use of immune checkpoint inhibitors in mesothelioma [Table 1]. Overall, the results have not been strongly successful, but several large studies have yet to report their data.

**Table 1:** Summary of completed and ongoing clinical trials on immune checkpoint inhibitors in mesothelioma.

Clinical trial	Phase	Intervention	Target	Control group	Number	ORR	Survival (months)
*Immune checkpoint inhibitors—monotherapy*							
MESOT-TREM-2008 (NCT01649024)	II	Tremelimumab	CTLA-4	None	29	7%	PFS 6.2, OS 10.7
MESOT-TREM-2012 (NCT01655888)	II	Tremelimumab	CTLA-4	None	29 (Est)	3.4%	PFS 6.2, OS 11.3
DETERMINE (NCT01843374)	IIb	Tremelimumab	CTLA-4	Placebo	571	NA	OS (treated group 7.7; placebo group 7.3)
KEYNOTE-028 (NCT02054806)	Ib	Pembrolizumab	PD-1	None	477	20%	PFS 5.4; OS 18
NCT02399371	II	Pembrolizumab	PD-1	None	65	pleural 20%, peritoneal 12.5%	PFS 4.5, OS 11.5
PROMISE-meso (NCT02991482)	III	Pembrolizumab	PD-1	Drug vs. gemcitabine or vinorelbine	144	treatment 22%, chemotherapy 6%	PFS (treatment 2.5, chemotherapy 3.4) OS (treatment 10.7, chemotherapy 11.7)
NCT02784171	II/III	Pembrolizumab	PD-1	Drug + pemetrexed/cisplatin vs pemetrexed/cisplatin	126 (Est)	NA: recruiting	
NCT04056026	I	Pembrolizumab + faecal microbiota transplant	PD-1		1	NA	
NCT02959463	I	Pembrolizumab + radiation therapy	PD-1	Hemithoracic radiation therapy + pembrolizumab; palliative radiotherapy + pembrolizumab	24 (Est)	NA: recruiting	
NCT02707666	I	Pembrolizumab	PD-1	Drug + surgery + pemetrexed/cisplatin	15 (Est)	NA: recruiting	
NIVOMES (NCT02497508)	I	Nivolumab	PD-1	None	33	24%	PFS 2.6; OS 11.8
MERIT (JapicCTI-163247)	II	Nivolumab	PD-1	None	34	29%	PFS 6.1; OS 17.3
CONFIRM (NCT03063450)	III	Nivolumab	PD-1	Control	336 (Est)	NA: recruiting	
JAVELIN (NCT01772004)	Ib	Avelumab	PD-L1	None	1758	9%	PFS 4.1; OS 10.7
NCT03399552	I/II	Avelumab + Stereotactic Body Radiation Therapy	PD-L1	None	27 (Est)	NA: recruiting	
DREAM	II	Durvalumab	PD-L1	Drug + pemetrexed & cisplatin + maintenance	54	48% (mRECIST) or 50% (iRECIST)	PFS 6.9
NCT02899195	II	Durvalumab	PD-L1	Drug + pemetrexed/ cisplatin vs concurrent + maintenance	55	NA: active, non-recruiting	
NCT03228537	I	Atezolizumab	PD-L1	Neoadjuvant + maintenance drug + surgery	28 (Est)	NA: recruiting	
*Combined immune checkpoint inhibitors*							
NCT02141347	I	Tremelimumab + Durvalumab	CTLA-4/PD-L1	None	65		
NIBIT-MESO-1 (NCT02588131)	II	Tremelimumab + Durvalumab	CTLA-4/PD-L1	None	40	28%	PFS 5.7; OS 16.6
NCT03075527	II	Tremelimumab + Durvalumab	CTLA-4/PD-L1	None	19	5%	PFS 2.8; OS 7.8
NCT02592551	II	Tremelimumab + Durvalumab	CTLA-4/PD-L1	Durvalumab, durvalumab + tremelimumab, vs placebo	20	NA: active, non-recruiting	
INITIATE (NCT03048474)	II	Ipilimumab + nivolumab	CTLA-4/PD-1	None	36	29%	PFS 6.2
MAPS-2 (NCT02716272)	II	Ipilimumab + nivolumab	CTLA-4/PD-1	Nivolumab vs nivolumab + ipilimumab	125	single 19%, dual 28%	PFS (single 4.0, dual 5.6) OS (single 11.9, dual 15.9)
Checkmate743 (NCT02899299)	III	Ipilimumab + nivolumab	CTLA-4/PD-1	Combination vs pemetrexed/cisplatin	606	NA: active, non-recruiting	
*Multimodal immunotherapy*							
NCT03393858	I/II	Autologous DCs + pembrolizumab + hyperthermia	PD-1		40 (Est)	NA: recruiting	
MESOVAX (NCT03546426)	I	Autologous DCs + pembrolizumab	PD-1	Autologous DCs + pembrolizumab + IL-2	18 (Est)	NA: not yet recruiting	
NCT04040231	I	Targeted cancer vaccine (WT1) + nivolumab	WT1/PD-1		10 (Est)	NA: recruiting	
NCT03126630	I/II	Anetumab ravtansine + Pembrolizumab	MSLN/PD-1	Pembrolizumab vs pembrolizumab + anetumab ravtansine	134 (Est)	NA: recruiting	
NCT03644550	II	LBM-100 + pembrolizumab	MSLN/PD-1	None	38 (Est)	NA: recruiting	
NCT03175172	II	CRS-207 + Pembrolizumab	MSLN/PD-1	None	10	Terminated (low enrolment; lack of clinical activity)	
NCT02758587	I/II	Defactinib + pembrolizumab	FAK/PD-1	None	59 (Est)	NA: recruiting	
NCT02414269	I	anti-MSLN CAR T cells + pembrolizumab	MSLN/PD-1	Drug + cyclophosphamide vs drug + pembrolizumab	66 (Est)	2/14 CR & 5/14 PR	
NCT03074513	II	Atezolizumab + Bevacizumab	PD-L1/VEGF	None	160 (Est)	NA: recruiting	

Figures for survival (in months) are represented by median values unless otherwise stated. ORR assessment criteria include the modified Response Evaluation Criteria in Solid Tumours for MPM (mRECIST) or RECIST modified for immunotherapy (iRECIST).

*CR* complete response, *CTLA-4* cytotoxic T-lymphocyte-associated protein 4, *DC* dendritic cell, *FAK* focal adhesion kinase, *IL-2* interleukin-2, *MSLN* mesothelin, *NA* not available, *ORR* overall response rate, *OS* overall survival, *PD-1* programmed cell death protein 1, *PD-L1* programmed death ligand 1, *PFS* progression-free survival, *PR* partial response, *VEGF* vascular endothelial growth factor, *WT1* Wilms’ tumour.

CTLA-4 appears not to be a useful target in mesothelioma. In an early trial, the anti-CTLA-4 monoclonal antibody (mAb) tremelimumab showed a limited response rate [115], whereas in a subsequent, more intensified, regimen, a partial response was seen in 1 of 29 patients enrolled in the study [116]. In a phase IIb trial, tremelimumab had no effect on OS [117]. Anti-PD-L1 approaches have yielded mixed results. In phase Ib and II trials, pembrolizumab, which targets the PD-1 receptor, appeared to improve overall response rate [118, 119], while increased PD-L1 expression was associated with a more durable PFS in the phase II study [119]. However, in a retrospective analysis, PD-L1 positivity was only a weak predictor of survival in pembrolizumab-treated cases [120]. In a phase III trial, pembrolizumab appears not to have improved overall survival (OS) compared with single-agent chemotherapy (ESMO 2019 conference, unpublished). Nivolumab, another anti-PD-1 mAb, slightly improved the overall response rate, with PD-L1 expression levels inconsistently predicting the response [121, 122]. A double-blind placebo-controlled trial (CONFIRM) to assess the effect of nivolumab on OS stopped recruitment last year but has yet to report results [123]. Other anti-PD-L1 agents including durvalumab and avelumab have also shown some promising activities [124, 125]. Theoretically, combining anti-CTLA4 with anti-PD-L1 treatment might be synergistic and avoid any potential emerging resistance. In a phase II trial, 40 mesothelioma patients given dual tremelimumab–durvalumab therapy showed a 28% partial response [126]. Likewise, combinations of ipilimumab plus nivolumab have been investigated in phase II trials; one trial failed to meet its primary endpoint [127], while another showed a 29% partial response [128]. In a randomised, open-label trial comparing nivolumab with nivolumab–ipilimumab dual therapy, the overall response rate was 19% with single and 29% with dual agents, but 5% of patients in the combination arm died from toxicity [129, 130]. The Checkmate743 phase III trial has now evaluated dual immunotherapy (nivolumab–ipilimumab) versus standard of care chemotherapy for patients with unresectable mesothelioma. Nivolumab plus ipilimumab significantly improved OS, predominantly in non-epithelioid disease (18.1 months versus 14.1 months for chemotherapy) [131]. The 2-year overall survival was 41% for nivolumab plus ipilimumab, but only 27% in the chemotherapy group.

Compared to other malignancies, mesothelioma is not highly immunogenic, which might explain its poor response to existing immune checkpoint inhibitors. Nevertheless, a number of proteins expressed by mesothelioma cells could serve as antigens for alternative immunotherapies, such as vaccines and CAR T cell therapy.

### Cancer vaccines

In 1982, immunotherapy for mesothelioma using the Bacillus Calmette–Guérin (BCG) vaccine was described in 30 patients, and resulted in apparent increased survival in a group of patients with low tumour burden. [132]. The implication was that the vaccine might stimulate the immune system to destroy tumour cells. The BCG vaccine is currently used to treat bladder cancer; it induces multiple cell types and cytokines including interleukins (IL-2, IL-4, IL-5, IL-6, IL-10, IL-12 and IL-17) and interferon (IFN)-γ [133]. IFN-β has anti-proliferative effects on mesothelioma cell lines in vitro [134] and, in patients, recombinant IFN-α triggered a partial response in 12% of recipients [135], while intrapleural IFN-γ showed a 20–45% response rate [136]. Anecdotal evidence has suggested that lymphokine-activated killer cells and IL-2 might reduce the formation of pleural effusions [137], and an early trial with IL-2 suggested survival benefit [138], with a subsequent phase II trial reporting tumour responses in 22% of patients with malignant mesothelioma [139]. These results have encouraged efforts to develop cancer vaccines and other immunotherapies that are more targeted to mesothelioma antigens.

#### Mesothelin as a target

Mesothelin is a 40-kDa cell surface glycoprotein expressed by all epithelioid, but not by sarcomatoid or the spindle component of biphasic mesotheliomas [140]. High levels of soluble mesothelin predict a poor prognosis, perhaps as a marker of tumour load [141]. Mesothelin-targeted therapies are under development. For instance, amatuximab, a mAb targeting mesothelin, showed acceptable safety profiles in early phase I trials [142] and triggered a partial response of 40% when combined with chemotherapy, improving overall survival in a phase II study [143]. CRS-207, a live attenuated form of *Listeria monocytogenes* engineered to express human mesothelin, stimulated a tumour-specific CD8^+^ T cell response in 60% of subjects with advanced cancer, including two patients with mesothelioma [144]. An early phase Ib trial of CRS-207 in combination with chemotherapy suggested an increased overall response rate, a reduction in tumour size, and the expansion of TILs and circulating immune cells [145]. However, a phase II trial of combined CRS-207 and pembrolizumab failed to show patient benefit [146]. SS1P, a recombinant protein that contains bacterial *Pseudomonas* exotoxin A fused to a high-affinity anti-mesothelin antibody, showed limited efficacy in phase I trials, possibly due to the development of neutralising antibodies, which occurred in most patients after a single treatment cycle [147]. A subsequent trial using concomitant cyclophosphamide and pentostatin to reduce antibody formation resulted in a response in 3 out of 10 patients [148]. Another anti-mesothelin immunotoxin, LMB-100, showed in vitro activity when additionally carrying a paclitaxel payload but has yet to be tested in patients [149].

#### WT1 as a target

As overexpression of the Wilms’ tumour 1 (WT1) transcription factor occurs in several malignancies, including mesothelioma [150], WT1 peptide analogue vaccines have been developed to elicit CD4^+^ and CD8^+^ T cell responses [151]. A favourable safety profile and potential therapeutic effect has been reported in patients randomised to receive a WT1-peptide vaccine (galinpepimut-S) with immunologic adjuvants (montanide and GM-CSF) compared with adjuvants alone [152].

#### Using tumour lysates

Following the success of the approach in murine models [153], dendritic cells pulsed with autologous tumour lysate were administered by vaccination to patients with mesothelioma in human trials [154]. This therapeutic approach was well-tolerated and induced cytotoxic activity in a subset of patients. In a subsequent trial, tumour-pulsed dendritic cells were combined with cyclophosphamide to enhance the immune response by inhibiting T_REG_ cells [155]. Only one patient achieved a complete response, but 7 of 10 patients survived for longer than 24 months. Autologous dendritic cells pulsed with allogeneic tumour lysate from 5 mesothelioma cell lines might also be effective [156]. A partial response was observed in 2 of 9 patients, although OS was unchanged [156]. A phase II/III trial (DENIM) using an allogeneic tumour-derived dendritic cell lysate vaccine (MesoPher) is currently recruiting adult subjects with mesothelioma (NCT03610360) [157].

### CAR T cell therapies

The immune system can also be engaged by generating chimeric antigen receptor (CAR) T cells. In this approach, homologous T cells are collected from the individual, genetically engineered to express a tumour-specific antigen receptor and, following ex vivo expansion, re-administered to the patient. The CAR consists of an antibody single-chain variable fragment (scFvs) fused to a transmembrane domain, followed by an intracellular co-stimulatory domain (including CD28, 4-1BB, CD27 and CD134) and an intracellular T cell receptor CD3ζ chain [158]. Following the success of this approach in haematological malignancies, the efficacy of CAR T cell therapy has been assessed in mesothelioma [159]. Originally, T cells were engineered to transiently express anti-mesothelin CAR to avoid off-tumour on-target toxicity [160]. A clinical response was attained in only 2 of 4 patients, and one patient suffered a cardiac arrest due to an anaphylactic reaction [161]. Several phase I studies have since attempted to optimise the safety profile of second-generation anti-mesothelin CAR T cells [162]. Of note, PD-1 expression in the tumour reduced CAR T cell effector function, but this effect could be reversed by PD-1 checkpoint blockade with pembrolizumab [163, 164]. CAR T cells can also be engineered to target peritumoural components such as fibroblast activation protein (FAP), a transmembrane serine protease highly expressed in cancer-associated stromal cells. Preliminary data using intrapleural FAP-targeted CAR T cells in mesothelioma showed redirected T cell activity in vitro with no significant toxicity [165]; these data were later supported by a phase I clinical trial involving three mesothelioma patients [166]. Other potential CAR T cell targets for mesothelioma include members of the ErbB family [167], oncofetal cell surface glycoprotein 5T4 and chondroitin sulphate proteoglycan 4 (CSPG4) [162, 168, 169].

### Antibody–drug conjugates

Antibody–drug conjugates (ADCs) use recombinant monoclonal antibodies that recognise tumour antigens to deliver cytotoxic payloads. Initially, as proof of concept, transferrin was used to deliver intracellular doxorubicin [170], but a number of ADCs that show activity in numerous cancers have subsequently been developed [171, 172]. Given its limited expression on normal tissues and high abundance on cancer cells, mesothelin is an attractive target [173]. So far, only one mesothelin-based ADC—BAY 94-9343 (anetumab ravtansine)—has been tested in mesothelioma. Anetumab ravtansine comprises a human anti-mesothelin antibody conjugated via a disulphide-containing linker to the maytansinoid DM4, which disrupts microtubule function and thus inhibits mitosis [174]. In vivo, anetumab ravtansine blocked mesothelioma growth in both subcutaneous and orthotopic xenograft models and was more effective than standard of care treatment [174]. Subsequently, three clinical trials have commenced for mesothelioma: a phase Ib trial of anetumab ravtansine in combination with pemetrexed and cisplatin (NCT02639091); a phase II study of anetumab ravtansine as a second-line treatment (NCT02610140); and a randomised phase I/II trial of anetumab ravtansine in combination with the anti-PD-1 mAb pembrolizumab (NCT03126630). However, anetumab ravtansine failed to increase PFS in relapsed mesothelioma in a phase II clinical trial (NCT02610140) when compared to vinorelbine, an anti-mitotic drug, [175].

Another ADC with potent anti-mesothelioma activity, αMSLN-MMAE, is a humanised anti-mesothelin mAb conjugated to the microtubule-disrupting drug monomethyl auristatin A (MMAE) with a lysosomal-protease-cleavable valine–citrulline linker [176]. A phase I clinical trial demonstrated that αMSLN–MMAE (also known as DMOT4039A) is well tolerated and exerts anti-tumour activity in patients with unresectable pancreatic or platinum-resistant ovarian cancer [177]; however, no clinical study of αMSLN-MMAE/DMOT4039A has yet been initiated for mesothelioma.

In vitro, epithelioid mesothelioma appears sensitive to brentuximab vedotin (BV), a recombinant chimeric mAb generated against CD30 and conjugated to MMAE via a protease-sensitive linker [178, 179]. Since CD30 is expressed to a greater degree in epithelioid than sarcomatoid mesotheliomas, studies will need to address if this agent shows subtype specificity. CD30 is a member of the tumour necrosis factor receptor (TNFR) superfamily that is involved in the regulation of apoptotic and inflammatory signalling pathways, and is a potential therapeutic target for various malignancies, including mesothelioma [180]. Brentuximab vedotin was initially tested for the treatment of anaplastic large-cell lymphomas and Hodgkin disease [181] but, following a successful safety assessment in solid tumours [182], a phase II trial is now underway to evaluate its efficacy in patients with unresectable CD30^+^ mesothelioma (NCT03007030).

A panel of ADCs that target trophoblast glycoprotein (also known as 5TA, an antigen expressed in several tumours) conjugated to a tubulin polymerisation inhibitor, yielded encouraging results in mesothelioma cells cultures expressing high levels of 5TA [183]. Another ADC was generated against the cell-surface glycoprotein CD26 [184], overexpressed in epithelioid and biphasic subtypes [185]. This ADC comprises the humanised anti-CD26 mAb YS110 and the TR-1 derivative of triptolide, a bioactive compound of *Tripterygium wilfordii* that shows a wide spectrum of anti-tumour activities [186]. It showed prominent cytotoxicity against mesothelioma and leukaemia cells in vitro and in vivo by impairing the RNA polymerase II activity through the TR-1-mediated inhibition of TFIIH, a transcription factor for RNA polymerase II [184].

### Oncolytic viral therapies

Oncolytic viruses offer another promising therapeutic approach for several malignancies, including mesothelioma [187, 188], through their dual anti-tumour activity involving the direct killing by lysis of infected cells (alongside the release of viral progeny to propagate the effect in neighbouring cells) and indirect induction of immune responses. The interaction between pathogen and host surface receptors triggers the production of type I IFNs, which leads to viral clearance and the release of tumour-associated antigens, as well as danger signals of cellular and viral origin. These factors all stimulate the expression of major histocompatibility complex (MHC) class I proteins and the recruitment of lymphoid cells, such as dendritic cells, CD4^+^ and CD8^+^ T cells, as well as NK cells [189]. Oncolytic viruses show an intrinsic selectivity towards tumour cells, which is mostly due to malignant characteristics such as altered metabolism, defects in anti-viral responses, the loss of p53 or p16 tumour suppressors and the activation of aberrant oncogenic signalling pathways, including RAS–MEK–ERK/MAPK and Wnt [188, 190, 191].

Thanks to its localised nature, relative lack of metastasis and its physical accessibility, mesothelioma is an attractive candidate for viral therapy. The intrapleural or intraperitoneal administration of double-stranded RNA was reported in 1976 to prevent tumour growth [192] and the successful targeted delivery of a viral construct was later confirmed in vitro and in vivo [193]. To date, several viruses, including adenoviruses [194], herpes simplex virus type 1 (HSV-1) [195], measles virus [196], vaccinia virus [197], Newcastle disease virus [198], retrovirus [199] and reovirus [200], have been tested in mesothelioma. A number of genetic modifications have been introduced to enhance the anti-tumour specificity and therapeutic efficacy. For instance, a VEGF-promoter-based adenovirus was shown to selectively replicate in mesothelioma cell lines; in xenograft mouse models, this construct suppressed tumour growth and prolonged animal survival [201]. The intra-tumoural delivery of retrovirus expressing the yeast cytosine deaminase prodrug activator gene was demonstrated to kill mesothelioma cells in vitro and to abolish tumour growth in vivo upon administration of the prodrug 5-fluorocytosine [199]. Furthermore, oncolytic adenovirus armed with a CD46-binding sequence and a promoter of heparin-binding growth factor midkine (both are highly expressed in mesothelioma cell lines) has been reported to confer enhanced infectivity and mesothelioma-specific cytotoxicity [202].

Another promising oncolytic virus for mesothelioma therapy is a replication-competent HSV in which the viral genes encoding ICP6 and ICP34.5 have been deleted. The lack of ICP6 confers virus selectivity towards mitotic cells or cells with a p16^INK4a^ deletion [203], suggesting a potential benefit in the treatment of mesothelioma with a loss of function of *CDKN2A*. Deletion of ICP34.5, a viral homologue of GADD34, markedly reduces the neurovirulence of the construct but inhibits viral protein synthesis [204], which consequently blunts viral replication and therapeutic efficacy. However, the homology of ICP34.5 to the DNA-damage-inducible GADD34 might enable viral replication to be complemented by the synergistic use of this construct with chemo- or radiotherapy [205, 206]; alternatively, defective viral replication could be overcome by introducing the *GADD34* gene into the HSV genome [207]. The latter approach might be particularly relevant for more aggressive histological subtypes of mesothelioma, which are characterised by a loss of GADD34 [8].

### Current clinical trials

Clinical trials investigating oncolytic viral therapy for mesothelioma are summarised in [Table 2]. The results from completed studies indicate that the adenovirus-mediated delivery of *IFNα2b* induced an anti-tumour immune response [208], which, when combined with chemotherapy as second-line treatment, significantly increased the OS [209]. When the clinical utility of vaccinia virus GL-ONC1 was assessed in patients with peritoneal cancers, including mesothelioma, tumour cell infection, virus replication and oncolysis were limited to the first cycle of treatment, possibly due to component-mediated virus inactivation as a result of the development of neutralising activities against GL-ONC1 [210]. The outcome of a phase II clinical trial investigating the effect of nivolumab in combination with MTG201, a replication-incompetent adenovirus containing the gene encoding REIC/Dkk-3, which confers anti-tumour activity, for mesothelioma is yet to be unveiled (NCT04013334).

**Table 2:** Summary of completed and ongoing clinical trials of oncolytic viral therapies in mesothelioma.

Identifier	Type of virus	Treatment mode	Integrated transgene/deletion	Status
NCT03710876	Adenovirus	rAd-IFN + celecoxib + gemcitabine	Human IFNα-2b	Recruiting
NCT04013334	Adenovirus	MTG201 + nivolumab	Immortalised cells (REIC)/Dikkopf (Dkk)-3	Recruiting
NCT01766739	Vaccinia virus	Monotherapy	β-galactosidase, β-glucuronidase, Ruc-GFP	Active, non-recruiting
NCT02714374	Vaccinia virus	GL-ONC1 −/+ eculizumab	β-galactosidase, β-glucuronidase, Ruc-GFP	Active, non-recruiting
NCT01997190	Adenovirus	AdV-tk + valacyclovir + chemotherapy	Herpes simplex virus thymidine kinase (HSV-TK)	Active, non-recruiting
NCT01503177	Measles virus	Monotherapy	Thyroidal sodium iodide symporter (NIS)	Active, non-recruiting
NCT02879669	Adenovirus	ONCOS-102 + pemetrexed/carboplatin	GM-CSF	Active, non-recruiting
NCT01569919	Vaccinia virus	TropVax + pemetrexed/cisplatin	TAA 5TA	Unknown
NCT01119664	Adenovirus	Ad.hIFNα2b + celecoxib + pemetrexed	IFNα2b	Completed
NCT00299962	Adenovirus	Monotherapy	IFNβ	Completed
NCT01212367	Adenovirus	Monotherapy	IFNα-2b	Completed
NCT01721018	HSV-I	Monotherapy	RL1 gene deletion encoding ICP34.5 protein	Completed

*GFP* green fluorescent protein, *GM-CSF* granulocyte-macrophage colony-stimulating factor, *HSV* Herpes simplex virus, *IFN* interferon, *REIC* reduced expression in immortalised cells, *TAA* tumour-associated antigen.

### Conclusions and future perspectives

Although studies of mesothelioma biology have revolutionised our understanding of this cancer, the prognosis for newly diagnosed patients nevertheless remains poor. The current epidemic of mesothelioma that is affecting industrialised countries will soon peak, but the number of cases is predicted to tail well into this century [211]. Unfortunately, the global consumption of asbestos remains undiminished owing to its growing use in middle-income nations, notably India and China [212]. Modern materials might also pose a future risk for mesothelioma: carbon nanotubes, for example, induce a mesothelioma-like pathology in preclinical models [213]. It is therefore important that clinical research focuses on mesothelioma.

**Fig. 3:**
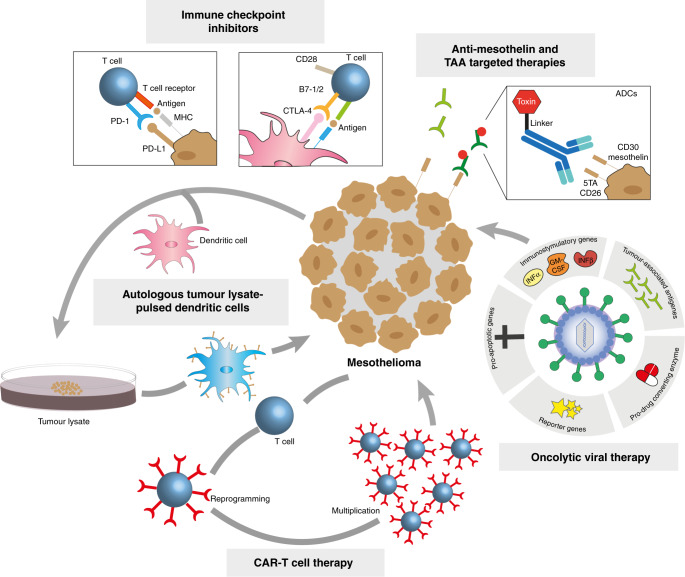
Therapeutic strategies in mesothelioma classified according to their mode of action. Immune checkpoint inhibitors block signalling that suppresses immune-cell activity, such as PD-1–PD-L1 and B7-1/2–CTLA-4 interactions, which is upregulated by tumour cells. Tumour-associated antigens (TAAs) can be targeted using monoclonal antibodies against proteins such as mesothelin, by vaccine therapy to stimulate the immune response and by using antibody–drug conjugates (ADCs) that target proteins such as mesothelin, 5TA, CD26 and CD30. Autologous dendritic cells pulsed with autologous or allogeneic tumour cell lysate act to prime host immunity, while chimeric antigen receptor CAR T cells contain chimaeric receptors that have been generated to specifically bind to TAAs on the cell surface. Other therapeutic approaches include oncolytic viruses that directly kill cancer cells by lysis or indirectly by stimulating immune response. They have been engineered to increase viral specificity (by introducing TAAs), cytotoxicity (by introducing e.g. pro-apoptotic or immunostimulatory genes), and monitoring (by introducing reporter genes).

Targeted therapies and immunotherapies that show promise in early trials frequently later fail in phase III trials. This might plausibly reflect a failure to pre-select those patients most likely to benefit from a particular treatment. Improved personalisation of therapies, through stratification of individuals by tumour type, antigen expression, or even genotype, might help overcome this [214]. There is cause for optimism in the treatment of mesothelioma. Innovative therapeutic approaches are now being directed towards mesothelioma [Fig. 3], while increased investment is enabling the development of better in vitro and in vivo model systems that should help to increase our chances of identifying effective therapies in the coming decade . It is crucial that this goal is achieved before the next wave of mesothelioma hits those countries that are currently repeating the mistakes of the West by consuming tonnes of asbestos to grow their economies.

**Fig. 4:**
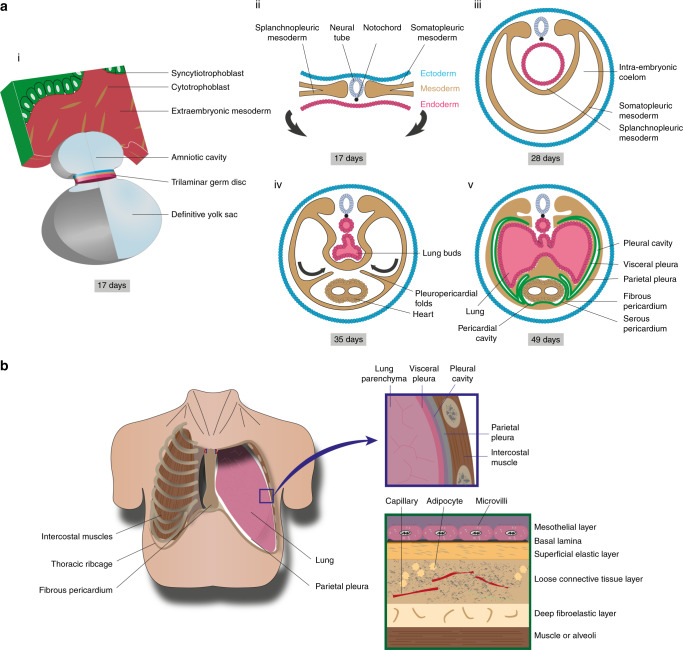
Pleural anatomy. **a** Embryonic pleural development. (i) Representation of the trilaminar germ disc following gastrulation together with the amniotic cavity and definitive yolk sac. (ii) During gastrulation, the mesoderm layer forms and the lateral plate mesoderm subdivides into somatic and splanchnic mesoderm. (iii) Following lateral flexion, the intra-embryonic coelom is lined by somatic and splanchnic mesoderm. (iv) The pleuropericardial folds extend to the midline and fuse with the ventral surface of foregut mesoderm, forming the primitive pleural cavities. (v) The somatic and splanchnic mesoderm is lined by parietal and visceral pleura respectively, which are contiguous with each other at the level of the hilum. **b** Anatomy of the thorax showing the relationship between the lungs, thoracic ribcage and pleura. The pleural cavity is defined by the space between adjacent visceral and parietal pleura. The pleura can be subdivided into five layers: mesothelial cells, basal lamina, superficial elastic layer, connective tissue layer and the deep fibroelastic layer. The deep layer is tightly adhered to the underlying structures e.g. muscle, rib or lung parenchyme.

## Data Availability

Not applicable.
